# Spontaneous Closure of Cyclodialysis Cleft in a Case of Normal-Tension Glaucoma Post Ab-Interno Trabeculotomy

**DOI:** 10.7759/cureus.23276

**Published:** 2022-03-17

**Authors:** Min-Yu Huang, Han-Yi Tseng

**Affiliations:** 1 Department of Ophthalmology, Kaohsiung Medical University Hospital, Kaohsiung, TWN; 2 Department of Ophthalmology, College of Medicine, Kaohsiung Medical University, Kaohsiung, TWN

**Keywords:** minimally invasive glaucoma surgery, glaucoma, normal tension glaucoma, spontaneous closure, cyclodialysis cleft

## Abstract

A cyclodialysis cleft (CDC) is the detachment of longitudinal ciliary muscle from scleral spur, causing an unusual communication between anterior chamber and suprachoroidal space, resulting in possible hypotony. We report a case of a 63-year-old woman with normal-tension glaucoma (NTG), who developed a shallow anterior chamber with relatively low intraocular pressure (IOP, 6-8 mmHg) after combined ab-interno trabeculotomy and phacoemulsification. Her vision reached good (20/20) with the mild myopic shift. After detecting subtle signs of clinical hypotony, CDC was confirmed and monitored using anterior segment optical coherence tomography. Five months after surgery, she encountered an episode of eye pain, with transient IOP elevation and deepening of the anterior chamber. Spontaneous closure of CDC was suspected and confirmed gonioscopically.

To the best of our knowledge, this is the first case to describe the clinical course of spontaneous CDC closure in a patient with NTG after ab-interno trabeculotomy. It is advisable to inform the patient about potential IOP spike after spontaneous closure once CDC is diagnosed.

## Introduction

A cyclodialysis cleft (CDC) is the detachment of longitudinal ciliary muscle from scleral spur, causing an unusual communication between anterior chamber and suprachoroidal space [1]. It usually occurs after blunt trauma or anterior segment surgery, leading to possible hypotony. We report a case of spontaneous closure of CDC in normal-tension glaucoma (NTG) after ab-interno trabeculotomy. To the best of our knowledge, this is the first case to report the clinical course of spontaneous CDC closure in a patient with NTG following ab-interno trabeculotomy.

## Case presentation

**Figure 1:**
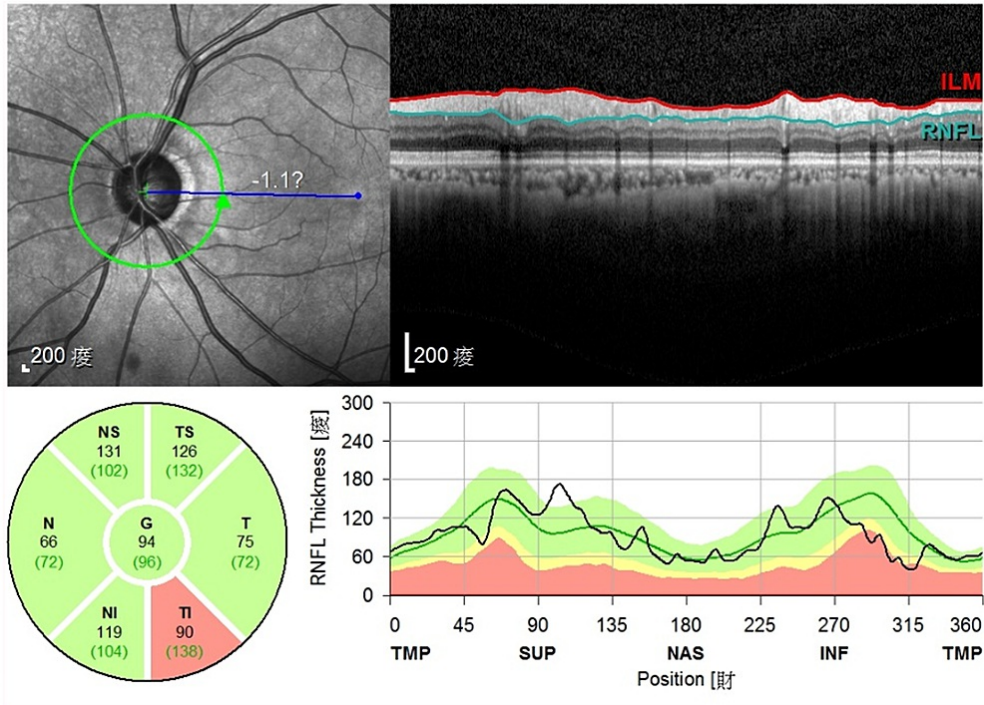
Optical coherence tomography of optic nerve head OCT showed loss of retinal nerve fiber layer, especially in the inferotemporal quadrant. OCT: optical coherence tomography; RNFL: retinal nerve fiber layer; ILM: internal limiting membrane; G: global; N: nasal; NI: nasal inferior; NS: nasal superior; T: temporal; TI: temporal inferior; TS: temporal superior; TMP: temporal; SUP: superior; NAS: nasal; INF: inferior

A healthy, 63-year-old woman with NTG on one medication, complained of increase in left eye blurring of vision (best-corrected visual acuity, BCVA, Snellen 20/60). Ocular exam revealed intraocular pressure (IOP) of 16 mmHg, nuclear sclerosis cataract, normal anterior segment and angle, enlarged disc cupping of 0.6, and normal fundus. Optical coherence tomography (OCT) showed loss of retinal nerve fiber layer (RNFL), especially in inferotemporal quadrant (Figure 1). After counseling, she opted for combined ab-interno trabeculotomy with phacoemulsification.

**Figure 2:**
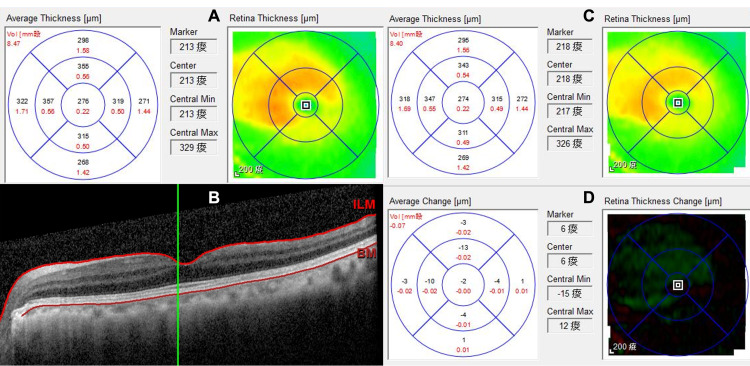
Optical coherence tomography of macula Before cleft closure: (A) slight thickening of the macula (B) no visible chorioretinal folds. After cleft closure: (C) reduction of macular thickening (D) change of retina thickness from A to C. OCT: optical coherence tomography; ILM: internal limiting membrane; BM: Bruch's membrane.

**Figure 3:**
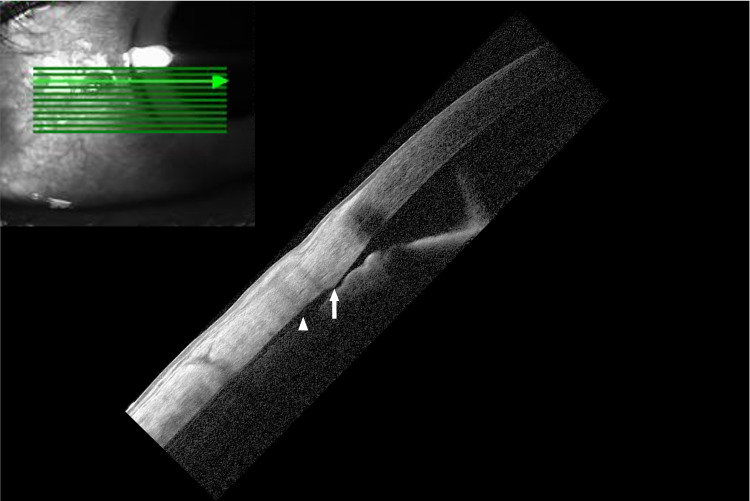
Anterior segment optical coherence tomography of nasal angle in left eye Cyclodialysis cleft (white arrow) and shallow suprachoroidal effusion (white arrowhead)

Ab-interno trabeculotomy was performed at nasal angle (7:00 to 10:00) using a spatula-shaped microhook (Inami & Co., Ltd, Tokyo, Japan) and the surgery was smooth intraoperatively. BCVA reached 20/20 at postoperative one week. However, IOP was on the low side (6-8 mmHg from postoperative one to four weeks). Ocular exam revealed a shallow anterior chamber (Grade 2 by Schaffer classification, no scleral spur visible) but the surgical wound was intact. There was a myopic shift but no visible fundus abnormalities. OCT showed slight thickening of macula without visible chorioretinal folds (Figures 2A-2B). Due to subtle signs of clinical hypotony (macular thickening), CDC was suspected and later confirmed by anterior segment OCT (AS-OCT) from 8:00 to 9:00 (Figure 3, white arrow), which had not been detected gonioscopically due to shallow anterior chamber.

**Figure 4:**
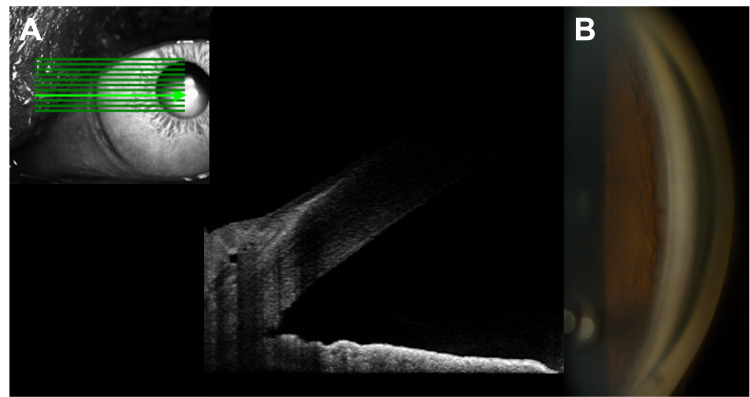
Closure of cyclodialysis cleft after IOP spike Closure of cyclodialysis cleft was suggested by (A) AS-OCT showing no apparent cleft and deep anterior chamber (B) wide-open nasal angle without visible cleft by gonioscopy. IOP: intraocular pressure; AS-OCT: anterior segment optical coherence tomography

To promote cleft closure, glaucoma medication was stopped together with early tapering of postoperative steroids. Due to stable vision, the patient was managed conservatively with topical cycloplegics. However, there was no change in IOP and the extent of the cleft on AS-OCT at 3-month follow-up. Five months after surgery, she encountered an episode of eye pain. IOP elevation to 42 mmHg was found in the emergency department, which was soon stabilized in the twenties by one medication and returned to 13 mmHg (with no medication) three weeks later. In subsequent clinic visits, she denied recent ocular trauma, steroid nor over the counter medication use. There were no signs of ocular inflammation. However, hyperopic shift and deepening of the anterior chamber were observed. OCT showed a reduction in macular thickening (Figures 2C-2D). Spontaneous closure of CDC was suspected because there was a deep anterior chamber without apparent cleft on AS-OCT (Figure 4A), together with a wide-opened nasal angle without visible cleft by gonioscopy (Figure 4B).

One year after surgery, despite IOP in the low teens, her visual field slightly progressed, and one topical medication was restarted for better IOP control.

## Discussion

With the increasing popularity of minimally invasive glaucoma surgery, iatrogenic CDCs are getting more common as more surgeons attempt to work at the angle [2]. However, the diagnosis and management of the CDC may be delayed due to the rarity of occurrence and the subtlety of clinical signs [3]. Our case illustrated three important points regarding the diagnosis and management of iatrogenic CDCs:

First, subtle signs of clinical hypotony. Despite not fulfilling the classical definition of hypotony (either numerical or clinical), our case presented with a myopic shift with shallow anterior chamber, suggesting anterior movement of intraocular lens with possible ciliary body detachment. Slight thickening of the macula on OCT was another important sign of subtle ocular hypotony (mild form of chorioretinal folds), leading us to the suspicion of CDC.

Second, increase diagnostic sensitivity of CDC by newer imaging modality. CDCs should be diagnosed and treated early to avoid the potential impact of ocular hypotony on vision [4]. However, visualization can be difficult in a soft eye or eyes with a shallow anterior chamber. Newer imaging modalities, such as AS-OCT in our case, serve as complementary tools to the diagnosis and monitoring of CDC [5].

Last but not least, IOP response after spontaneous CDC closure in NTG. There is currently little evidence that whether CDC closure in NTG shares a similar IOP response as in high tension glaucoma. Our patient experienced an episode of eye pain followed by IOP elevation, which was even higher than preoperative IOP. After excluding other causes for IOP elevation, such as ocular trauma, steroid response, or ocular inflammation, spontaneous closure of CDC was suspected due to IOP elevation with deepening of the anterior chamber. Despite the mechanism remains unclear, IOP spike after spontaneous CDC closure was reported [2]. It was hypothesized that post-closure IOP spike occurs due to the collapse of drainage channels [6]. The IOP may become normalized again after re-opening of drainage channels, as in our case or previous reports [2,3,6]. We believe that the better IOP control in our patient after pressure spike may be related to the effect of previous ab-interno trabeculotomy, like in the previous report [2]. Although a post-closure IOP spike was observed in our case, it requires further study to explore whether this can be generalized to other NTG eyes. However, it is advisable to counsel the patient about potential IOP spike after spontaneous closure once CDC is diagnosed, as well as the importance of regular follow-up.

## Conclusions

CDC is rare, and the diagnosis may be delayed due to low suspicion and subtle clinical signs. In the absence of significant ocular hypotony, newer imaging modalities may help to support the diagnosis both directly and indirectly. To the best of our knowledge, our case is the first to describe the clinical course of spontaneous CDC closure in a patient with NTG after ab-interno trabeculotomy. It is advisable to inform the patient about potential IOP spike after spontaneous closure once CDC is diagnosed, as well as the importance of regular follow-up.
